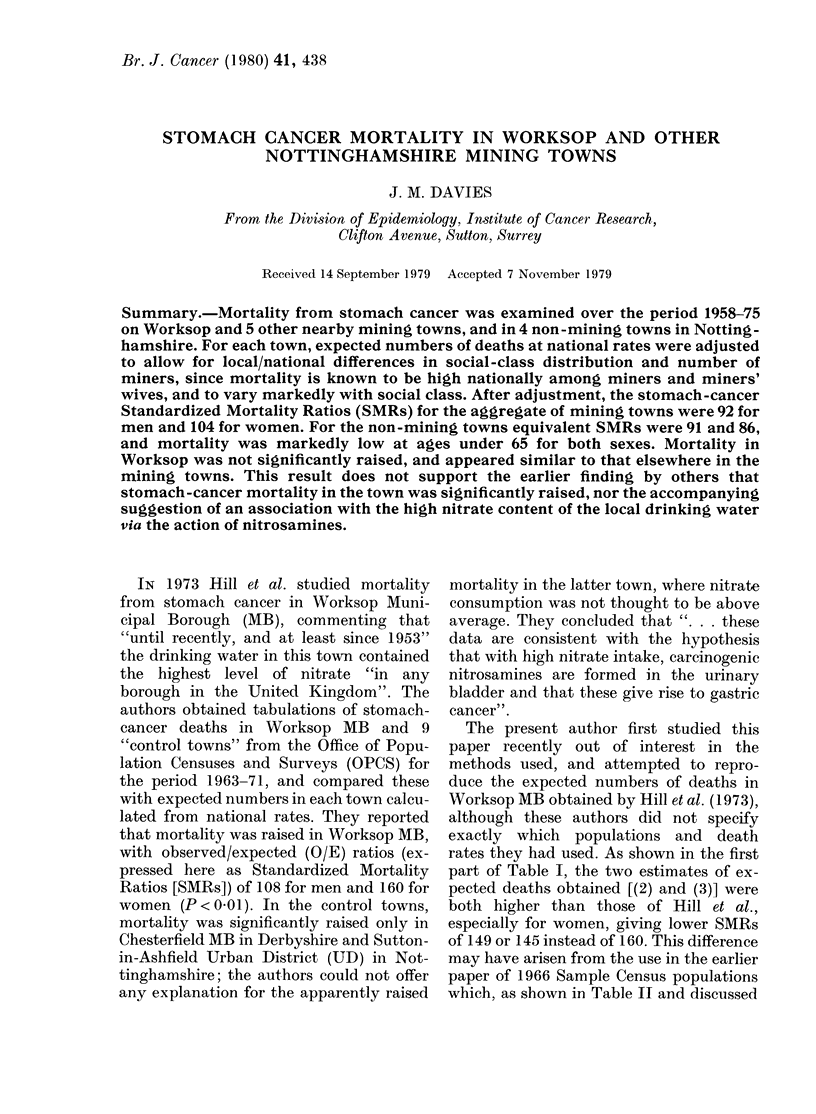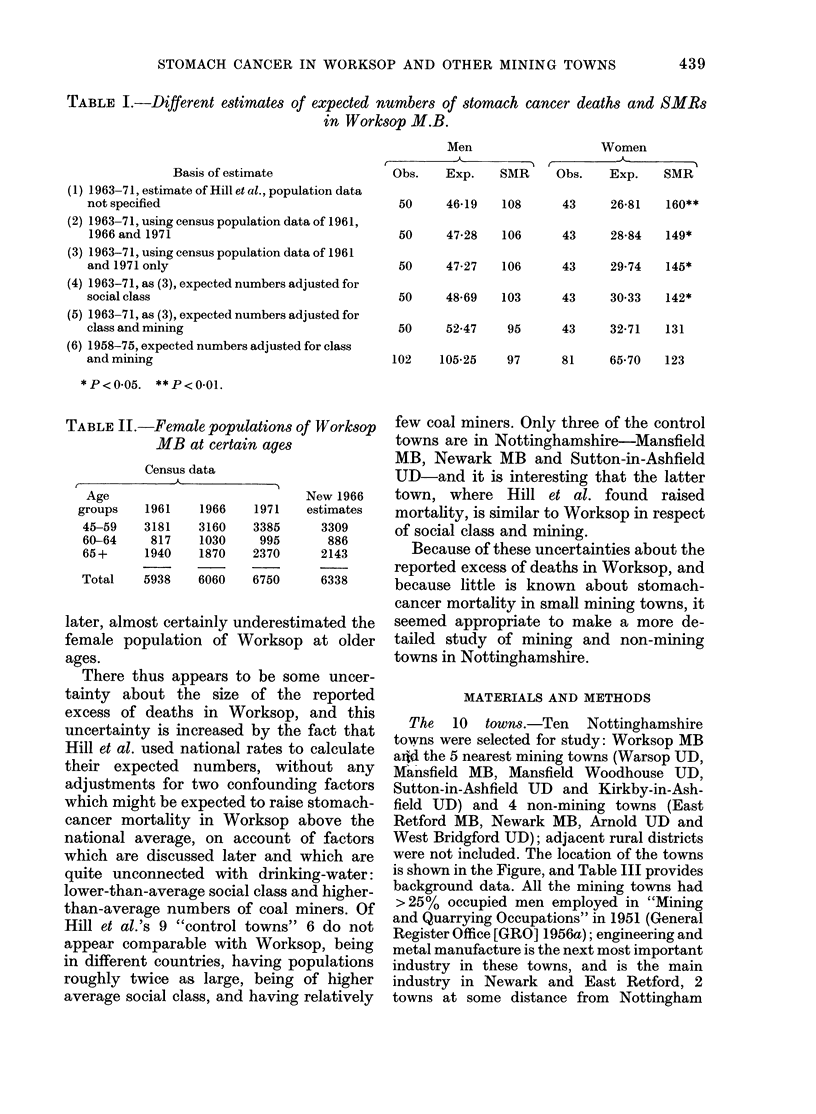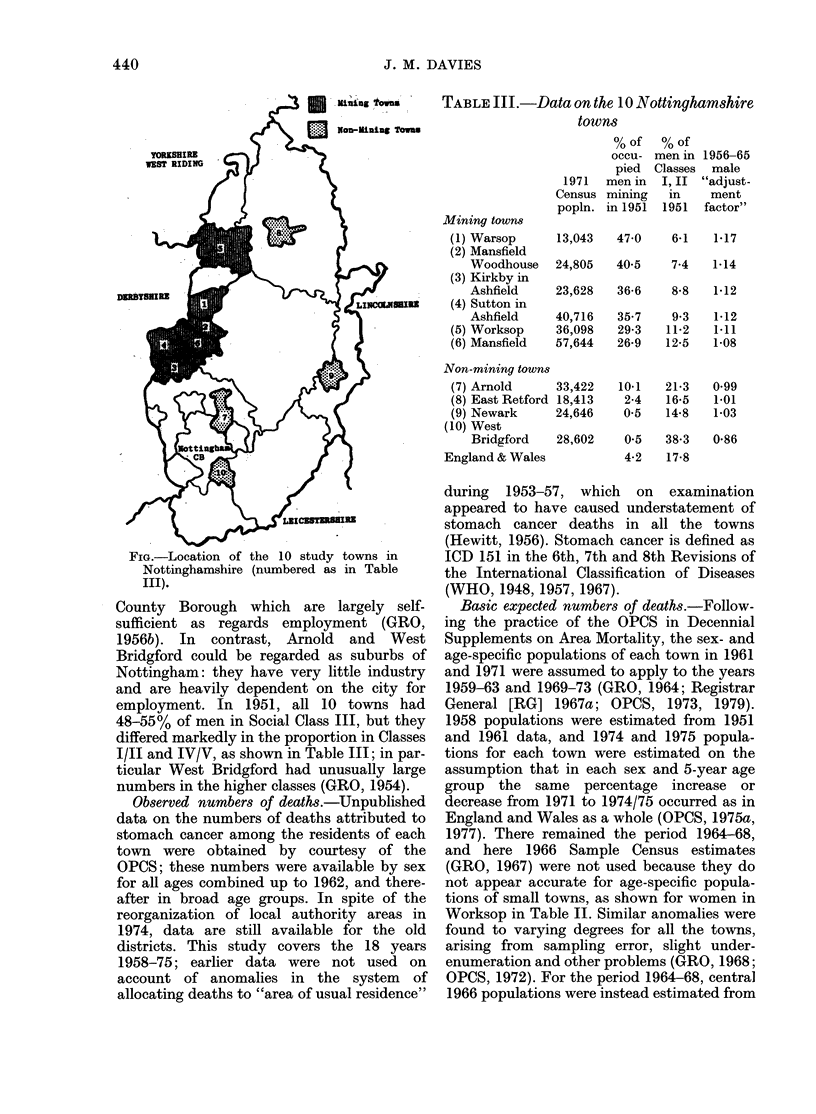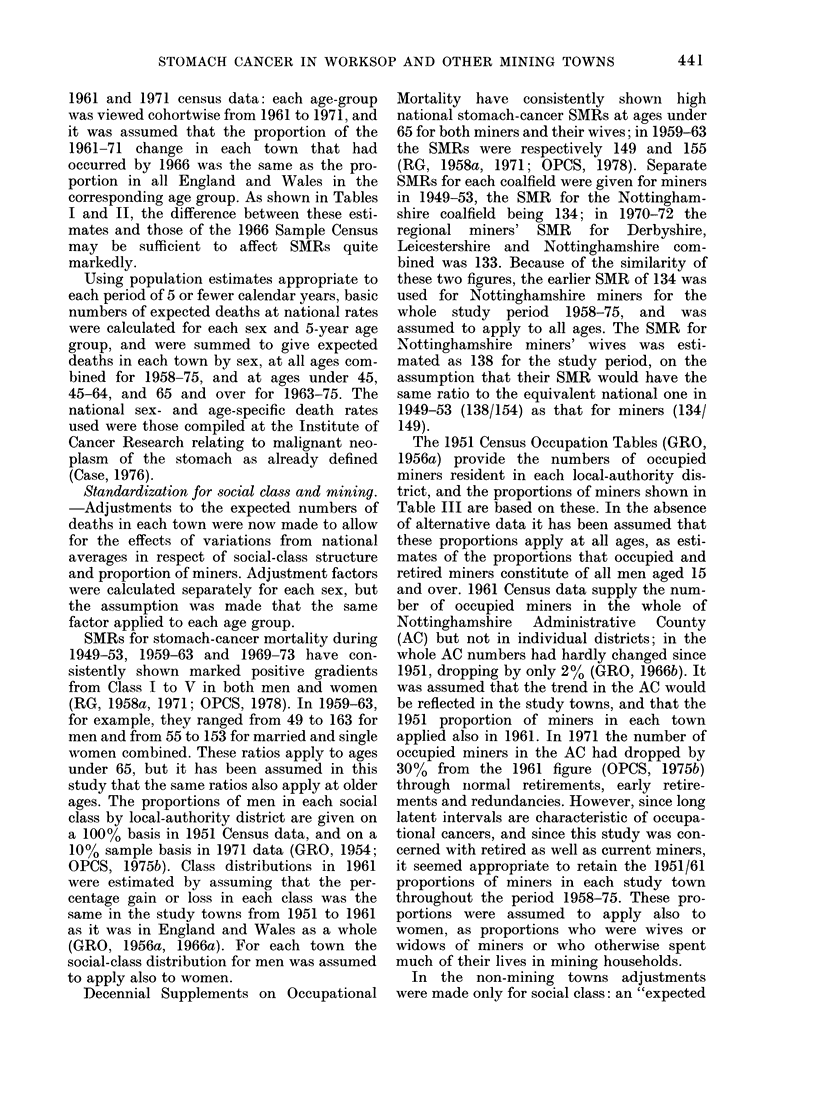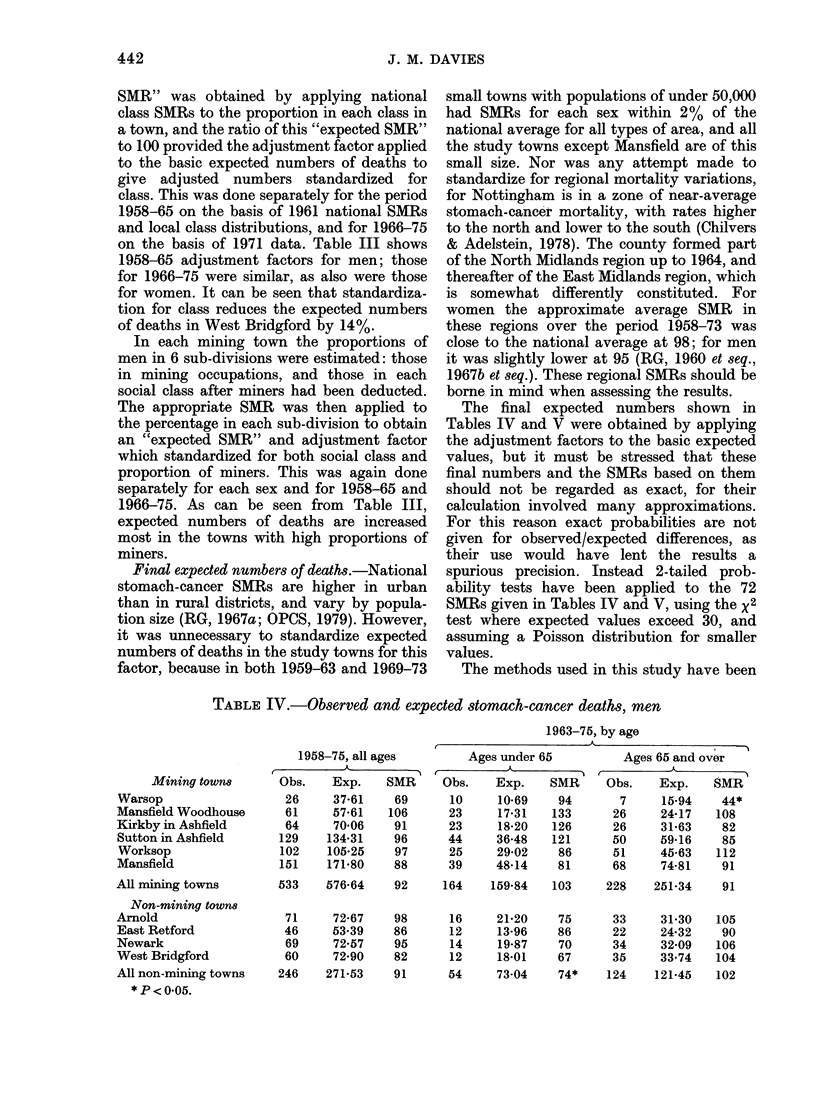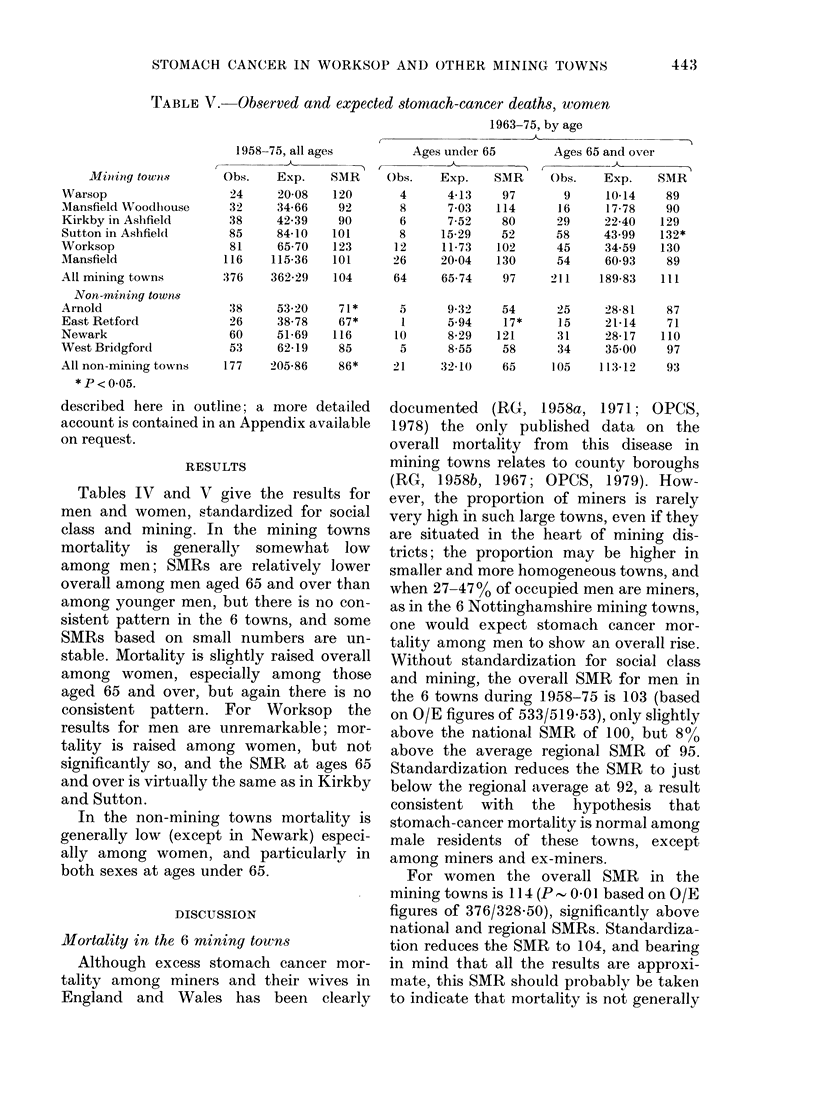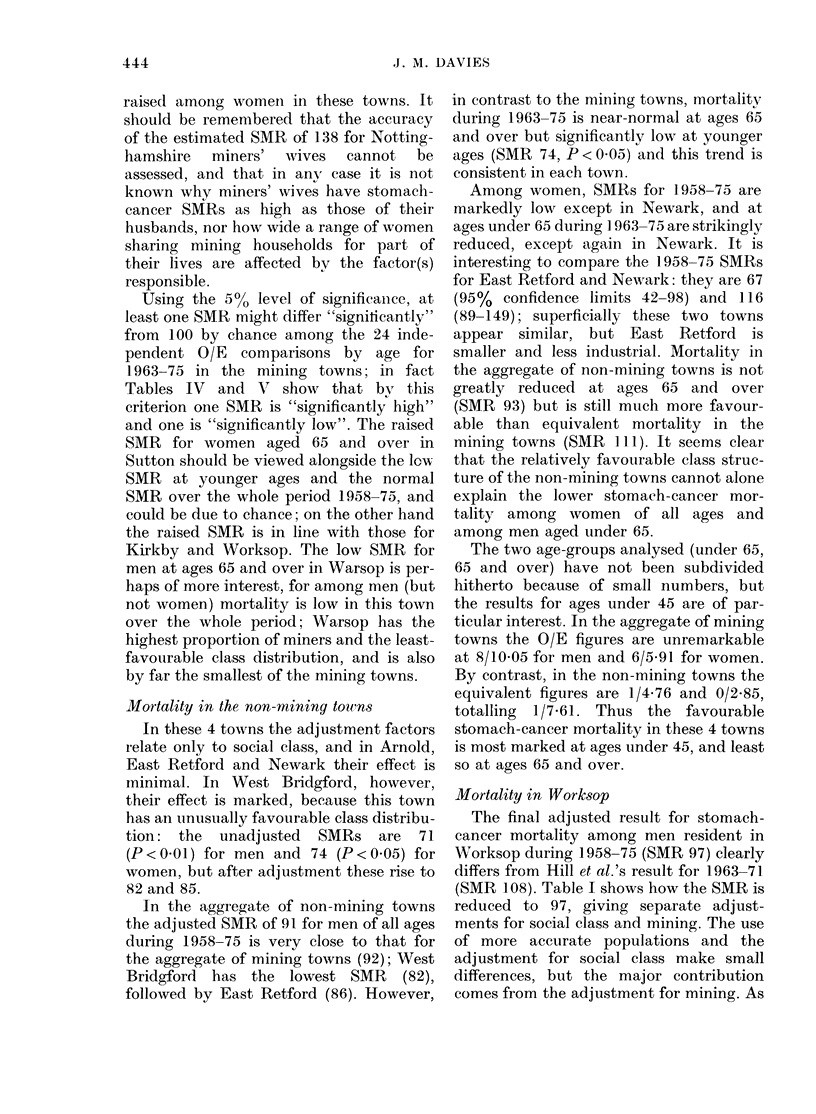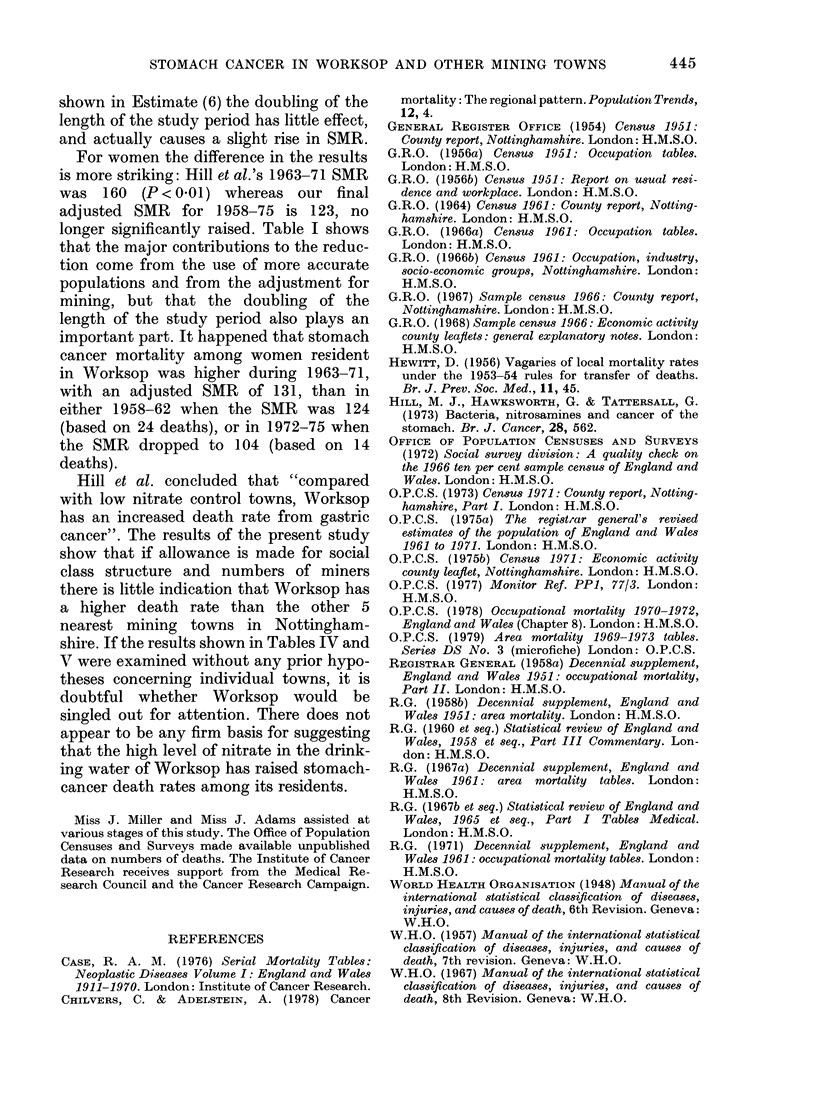# Stomach cancer mortality in Worksop and other Nottinghamshire mining towns.

**DOI:** 10.1038/bjc.1980.68

**Published:** 1980-03

**Authors:** J. M. Davies

## Abstract

Mortality from stomach cancer was examined over the period 1958-75 on Workshop and 5 other nearby mining towns, and in 4 non-mining towns in Nottinghamshire. For each town, expected numbers of deaths at national rates were adjusted to allow for local/national differences in social-class distribution and number of miners, since mortality is known to be high nationally among miners and miners' wives, and to vary markedly with social class. After adjustment, the stomach-cancer Standardized Mortality Ratios (SMRs) for the aggregate of mining towns were 92 for men and 104 for women. For the non-mining towns equivalent SMRs were 91 and 86, and mortality was markedly low at ages under 65 for both sexes. Mortality in Workshop was not significantly raised, and appeared similar to that elsewhere in the mining towns. This result does not support the earlier finding by others that stomach-cancer mortality in the town was significantly raised, nor the accompanying suggestion of an association with the high nitrate content of the local drinking water via the action of nitrosamines.


*Br. J. Cancer* (1980) **41**, 438

### STOMACH CANCER MORTALITY IN WORKSOP AND OTHER NOTTINGHAMSHIRE MINING TOWNS

J. M. DAVIES

*From the Division of Epidemiology, Institute of Cancer Research, Clifton Avenue, Sutton, Surrey*

Received 14 September 1979 Accepted 7 November 1979

**Summary.—Mortality from stomach cancer was examined over the period 1958–75 on Worksop and 5 other nearby mining towns, and in 4 non-mining towns in Nottinghamshire. For each town, expected numbers of deaths at national rates were adjusted to allow for local/national differences in social-class distribution and number of miners, since mortality is known to be high nationally among miners and miners' wives, and to vary markedly with social class. After adjustment, the stomach-cancer Standardized Mortality Ratios (SMRs) for the aggregate of mining towns were 92 for men and 104 for women. For the non-mining towns equivalent SMRs were 91 and 86, and mortality was markedly low at ages under 65 for both sexes. Mortality in Worksop was not significantly raised, and appeared similar to that elsewhere in the mining towns. This result does not support the earlier finding by others that stomach-cancer mortality in the town was significantly raised, nor the accompanying suggestion of an association with the high nitrate content of the local drinking water *via* the action of nitrosamines.**

IN 1973 Hill *et al.* studied mortality from stomach cancer in Worksop Municipal Borough (MB), commenting that "until recently, and at least since 1953" the drinking water in this town contained the highest level of nitrate "in any borough in the United Kingdom". The authors obtained tabulations of stomach-cancer deaths in Worksop MB and 9 "control towns" from the Office of Population Censuses and Surveys (OPCS) for the period 1963–71, and compared these with expected numbers in each town calculated from national rates. They reported that mortality was raised in Worksop MB, with observed/expected (O/E) ratios (expressed here as Standardized Mortality Ratios [SMRs]) of 108 for men and 160 for women ($P < 0{\cdot}01$). In the control towns, mortality was significantly raised only in Chesterfield MB in Derbyshire and Sutton-in-Ashfield Urban District (UD) in Nottinghamshire; the authors could not offer any explanation for the apparently raised mortality in the latter town, where nitrate consumption was not thought to be above average. They concluded that ". . . these data are consistent with the hypothesis that with high nitrate intake, carcinogenic nitrosamines are formed in the urinary bladder and that these give rise to gastric cancer".

The present author first studied this paper recently out of interest in the methods used, and attempted to reproduce the expected numbers of deaths in Worksop MB obtained by Hill *et al.* (1973), although these authors did not specify exactly which populations and death rates they had used. As shown in the first part of Table I, the two estimates of expected deaths obtained [(2) and (3)] were both higher than those of Hill *et al.*, especially for women, giving lower SMRs of 149 or 145 instead of 160. This difference may have arisen from the use in the earlier paper of 1966 Sample Census populations which, as shown in Table II and discussed

TABLE I.—*Different estimates of expected numbers of stomach cancer deaths and SMRs in Worksop M.B.*

| Basis of estimate | Men | | | Women | | |
|---|---|---|---|---|---|---|
| | Obs. | Exp. | SMR | Obs. | Exp. | SMR |
| (1) 1963–71, estimate of Hill *et al.*, population data not specified | 50 | 46·19 | 108 | 43 | 26·81 | 160** |
| (2) 1963–71, using census population data of 1961, 1966 and 1971 | 50 | 47·28 | 106 | 43 | 28·84 | 149* |
| (3) 1963–71, using census population data of 1961 and 1971 only | 50 | 47·27 | 106 | 43 | 29·74 | 145* |
| (4) 1963–71, as (3), expected numbers adjusted for social class | 50 | 48·69 | 103 | 43 | 30·33 | 142* |
| (5) 1963–71, as (3), expected numbers adjusted for class and mining | 50 | 52·47 | 95 | 43 | 32·71 | 131 |
| (6) 1958–75, expected numbers adjusted for class and mining | 102 | 105·25 | 97 | 81 | 65·70 | 123 |

* $P < 0·05$. ** $P < 0·01$.

TABLE II.—*Female populations of Worksop MB at certain ages*

| Age groups | Census data 1961 | 1966 | 1971 | New 1966 estimates |
|---|---|---|---|---|
| 45–59 | 3181 | 3160 | 3385 | 3309 |
| 60–64 | 817 | 1030 | 995 | 886 |
| 65+ | 1940 | 1870 | 2370 | 2143 |
| Total | 5938 | 6060 | 6750 | 6338 |

later, almost certainly underestimated the female population of Worksop at older ages.

There thus appears to be some uncertainty about the size of the reported excess of deaths in Worksop, and this uncertainty is increased by the fact that Hill *et al.* used national rates to calculate their expected numbers, without any adjustments for two confounding factors which might be expected to raise stomach-cancer mortality in Worksop above the national average, on account of factors which are discussed later and which are quite unconnected with drinking-water: lower-than-average social class and higher-than-average numbers of coal miners. Of Hill *et al.*'s 9 "control towns" 6 do not appear comparable with Worksop, being in different countries, having populations roughly twice as large, being of higher average social class, and having relatively few coal miners. Only three of the control towns are in Nottinghamshire—Mansfield MB, Newark MB and Sutton-in-Ashfield UD—and it is interesting that the latter town, where Hill *et al.* found raised mortality, is similar to Worksop in respect of social class and mining.

Because of these uncertainties about the reported excess of deaths in Worksop, and because little is known about stomach-cancer mortality in small mining towns, it seemed appropriate to make a more detailed study of mining and non-mining towns in Nottinghamshire.

### MATERIALS AND METHODS

*The* 10 *towns.*—Ten Nottinghamshire towns were selected for study: Worksop MB and the 5 nearest mining towns (Warsop UD, Mansfield MB, Mansfield Woodhouse UD, Sutton-in-Ashfield UD and Kirkby-in-Ashfield UD) and 4 non-mining towns (East Retford MB, Newark MB, Arnold UD and West Bridgford UD); adjacent rural districts were not included. The location of the towns is shown in the Figure, and Table III provides background data. All the mining towns had >25% occupied men employed in "Mining and Quarrying Occupations" in 1951 (General Register Office [GRO] 1956*a*); engineering and metal manufacture is the next most important industry in these towns, and is the main industry in Newark and East Retford, 2 towns at some distance from Nottingham

FIG.—Location of the 10 study towns in Nottinghamshire (numbered as in Table III).

County Borough which are largely self-sufficient as regards employment (GRO, 1956*b*). In contrast, Arnold and West Bridgford could be regarded as suburbs of Nottingham: they have very little industry and are heavily dependent on the city for employment. In 1951, all 10 towns had 48–55% of men in Social Class III, but they differed markedly in the proportion in Classes I/II and IV/V, as shown in Table III; in particular West Bridgford had unusually large numbers in the higher classes (GRO, 1954).

*Observed numbers of deaths.*—Unpublished data on the numbers of deaths attributed to stomach cancer among the residents of each town were obtained by courtesy of the OPCS; these numbers were available by sex for all ages combined up to 1962, and thereafter in broad age groups. In spite of the reorganization of local authority areas in 1974, data are still available for the old districts. This study covers the 18 years 1958–75; earlier data were not used on account of anomalies in the system of allocating deaths to "area of usual residence" during 1953–57, which on examination appeared to have caused understatement of stomach cancer deaths in all the towns (Hewitt, 1956). Stomach cancer is defined as ICD 151 in the 6th, 7th and 8th Revisions of the International Classification of Diseases (WHO, 1948, 1957, 1967).

TABLE III.—*Data on the* 10 *Nottinghamshire towns*

| | 1971 Census popln. | % of occupied men in mining in 1951 | % of men in Classes I, II in 1951 | 1956–65 male "adjustment factor" |
|---|---|---|---|---|
| *Mining towns* | | | | |
| (1) Warsop | 13,043 | 47·0 | 6·1 | 1·17 |
| (2) Mansfield Woodhouse | 24,805 | 40·5 | 7·4 | 1·14 |
| (3) Kirkby in Ashfield | 23,628 | 36·6 | 8·8 | 1·12 |
| (4) Sutton in Ashfield | 40,716 | 35·7 | 9·3 | 1·12 |
| (5) Worksop | 36,098 | 29·3 | 11·2 | 1·11 |
| (6) Mansfield | 57,644 | 26·9 | 12·5 | 1·08 |
| *Non-mining towns* | | | | |
| (7) Arnold | 33,422 | 10·1 | 21·3 | 0·99 |
| (8) East Retford | 18,413 | 2·4 | 16·5 | 1·01 |
| (9) Newark | 24,646 | 0·5 | 14·8 | 1·03 |
| (10) West Bridgford | 28,602 | 0·5 | 38·3 | 0·86 |
| England & Wales | | 4·2 | 17·8 | |

*Basic expected numbers of deaths.*—Following the practice of the OPCS in Decennial Supplements on Area Mortality, the sex- and age-specific populations of each town in 1961 and 1971 were assumed to apply to the years 1959–63 and 1969–73 (GRO, 1964; Registrar General [RG] 1967*a*; OPCS, 1973, 1979). 1958 populations were estimated from 1951 and 1961 data, and 1974 and 1975 populations for each town were estimated on the assumption that in each sex and 5-year age group the same percentage increase or decrease from 1971 to 1974/75 occurred as in England and Wales as a whole (OPCS, 1975*a*, 1977). There remained the period 1964–68, and here 1966 Sample Census estimates (GRO, 1967) were not used because they do not appear accurate for age-specific populations of small towns, as shown for women in Worksop in Table II. Similar anomalies were found to varying degrees for all the towns, arising from sampling error, slight under-enumeration and other problems (GRO, 1968; OPCS, 1972). For the period 1964–68, central 1966 populations were instead estimated from

1961 and 1971 census data: each age-group was viewed cohortwise from 1961 to 1971, and it was assumed that the proportion of the 1961–71 change in each town that had occurred by 1966 was the same as the proportion in all England and Wales in the corresponding age group. As shown in Tables I and II, the difference between these estimates and those of the 1966 Sample Census may be sufficient to affect SMRs quite markedly.

Using population estimates appropriate to each period of 5 or fewer calendar years, basic numbers of expected deaths at national rates were calculated for each sex and 5-year age group, and were summed to give expected deaths in each town by sex, at all ages combined for 1958–75, and at ages under 45, 45–64, and 65 and over for 1963–75. The national sex- and age-specific death rates used were those compiled at the Institute of Cancer Research relating to malignant neoplasm of the stomach as already defined (Case, 1976).

*Standardization for social class and mining.*—Adjustments to the expected numbers of deaths in each town were now made to allow for the effects of variations from national averages in respect of social-class structure and proportion of miners. Adjustment factors were calculated separately for each sex, but the assumption was made that the same factor applied to each age group.

SMRs for stomach-cancer mortality during 1949–53, 1959–63 and 1969–73 have consistently shown marked positive gradients from Class I to V in both men and women (RG, 1958*a*, 1971; OPCS, 1978). In 1959–63, for example, they ranged from 49 to 163 for men and from 55 to 153 for married and single women combined. These ratios apply to ages under 65, but it has been assumed in this study that the same ratios also apply at older ages. The proportions of men in each social class by local-authority district are given on a 100% basis in 1951 Census data, and on a 10% sample basis in 1971 data (GRO, 1954; OPCS, 1975*b*). Class distributions in 1961 were estimated by assuming that the percentage gain or loss in each class was the same in the study towns from 1951 to 1961 as it was in England and Wales as a whole (GRO, 1956*a*, 1966*a*). For each town the social-class distribution for men was assumed to apply also to women.

Decennial Supplements on Occupational Mortality have consistently shown high national stomach-cancer SMRs at ages under 65 for both miners and their wives; in 1959–63 the SMRs were respectively 149 and 155 (RG, 1958*a*, 1971; OPCS, 1978). Separate SMRs for each coalfield were given for miners in 1949–53, the SMR for the Nottinghamshire coalfield being 134; in 1970–72 the regional miners' SMR for Derbyshire, Leicestershire and Nottinghamshire combined was 133. Because of the similarity of these two figures, the earlier SMR of 134 was used for Nottinghamshire miners for the whole study period 1958–75, and was assumed to apply to all ages. The SMR for Nottinghamshire miners' wives was estimated as 138 for the study period, on the assumption that their SMR would have the same ratio to the equivalent national one in 1949–53 (138/154) as that for miners (134/149).

The 1951 Census Occupation Tables (GRO, 1956*a*) provide the numbers of occupied miners resident in each local-authority district, and the proportions of miners shown in Table III are based on these. In the absence of alternative data it has been assumed that these proportions apply at all ages, as estimates of the proportions that occupied and retired miners constitute of all men aged 15 and over. 1961 Census data supply the number of occupied miners in the whole of Nottinghamshire Administrative County (AC) but not in individual districts; in the whole AC numbers had hardly changed since 1951, dropping by only 2% (GRO, 1966*b*). It was assumed that the trend in the AC would be reflected in the study towns, and that the 1951 proportion of miners in each town applied also in 1961. In 1971 the number of occupied miners in the AC had dropped by 30% from the 1961 figure (OPCS, 1975*b*) through normal retirements, early retirements and redundancies. However, since long latent intervals are characteristic of occupational cancers, and since this study was concerned with retired as well as current miners, it seemed appropriate to retain the 1951/61 proportions of miners in each study town throughout the period 1958–75. These proportions were assumed to apply also to women, as proportions who were wives or widows of miners or who otherwise spent much of their lives in mining households.

In the non-mining towns adjustments were made only for social class: an "expected

SMR" was obtained by applying national class SMRs to the proportion in each class in a town, and the ratio of this "expected SMR" to 100 provided the adjustment factor applied to the basic expected numbers of deaths to give adjusted numbers standardized for class. This was done separately for the period 1958–65 on the basis of 1961 national SMRs and local class distributions, and for 1966–75 on the basis of 1971 data. Table III shows 1958–65 adjustment factors for men; those for 1966–75 were similar, as also were those for women. It can be seen that standardization for class reduces the expected numbers of deaths in West Bridgford by 14%.

In each mining town the proportions of men in 6 sub-divisions were estimated: those in mining occupations, and those in each social class after miners had been deducted. The appropriate SMR was then applied to the percentage in each sub-division to obtain an "expected SMR" and adjustment factor which standardized for both social class and proportion of miners. This was again done separately for each sex and for 1958–65 and 1966–75. As can be seen from Table III, expected numbers of deaths are increased most in the towns with high proportions of miners.

*Final expected numbers of deaths.*—National stomach-cancer SMRs are higher in urban than in rural districts, and vary by population size (RG, 1967*a*; OPCS, 1979). However, it was unnecessary to standardize expected numbers of deaths in the study towns for this factor, because in both 1959–63 and 1969–73 small towns with populations of under 50,000 had SMRs for each sex within 2% of the national average for all types of area, and all the study towns except Mansfield are of this small size. Nor was any attempt made to standardize for regional mortality variations, for Nottingham is in a zone of near-average stomach-cancer mortality, with rates higher to the north and lower to the south (Chilvers & Adelstein, 1978). The county formed part of the North Midlands region up to 1964, and thereafter of the East Midlands region, which is somewhat differently constituted. For women the approximate average SMR in these regions over the period 1958–73 was close to the national average at 98; for men it was slightly lower at 95 (RG, 1960 *et seq.*, 1967*b et seq.*). These regional SMRs should be borne in mind when assessing the results.

The final expected numbers shown in Tables IV and V were obtained by applying the adjustment factors to the basic expected values, but it must be stressed that these final numbers and the SMRs based on them should not be regarded as exact, for their calculation involved many approximations. For this reason exact probabilities are not given for observed/expected differences, as their use would have lent the results a spurious precision. Instead 2-tailed probability tests have been applied to the 72 SMRs given in Tables IV and V, using the $\chi^2$ test where expected values exceed 30, and assuming a Poisson distribution for smaller values.

The methods used in this study have been

TABLE IV.—*Observed and expected stomach-cancer deaths, men*

| | 1958–75, all ages | | | 1963–75, by age: Ages under 65 | | | 1963–75, by age: Ages 65 and over | | |
|---|---|---|---|---|---|---|---|---|---|
| *Mining towns* | Obs. | Exp. | SMR | Obs. | Exp. | SMR | Obs. | Exp. | SMR |
| Warsop | 26 | 37·61 | 69 | 10 | 10·69 | 94 | 7 | 15·94 | 44* |
| Mansfield Woodhouse | 61 | 57·61 | 106 | 23 | 17·31 | 133 | 26 | 24·17 | 108 |
| Kirkby in Ashfield | 64 | 70·06 | 91 | 23 | 18·20 | 126 | 26 | 31·63 | 82 |
| Sutton in Ashfield | 129 | 134·31 | 96 | 44 | 36·48 | 121 | 50 | 59·16 | 85 |
| Worksop | 102 | 105·25 | 97 | 25 | 29·02 | 86 | 51 | 45·63 | 112 |
| Mansfield | 151 | 171·80 | 88 | 39 | 48·14 | 81 | 68 | 74·81 | 91 |
| All mining towns | 533 | 576·64 | 92 | 164 | 159·84 | 103 | 228 | 251·34 | 91 |
| *Non-mining towns* | | | | | | | | | |
| Arnold | 71 | 72·67 | 98 | 16 | 21·20 | 75 | 33 | 31·30 | 105 |
| East Retford | 46 | 53·39 | 86 | 12 | 13·96 | 86 | 22 | 24·32 | 90 |
| Newark | 69 | 72·57 | 95 | 14 | 19·87 | 70 | 34 | 32·09 | 106 |
| West Bridgford | 60 | 72·90 | 82 | 12 | 18·01 | 67 | 35 | 33·74 | 104 |
| All non-mining towns | 246 | 271·53 | 91 | 54 | 73·04 | 74* | 124 | 121·45 | 102 |

* $P < 0·05$.

TABLE V.—*Observed and expected stomach-cancer deaths, women*

| | 1958–75, all ages | | | 1963–75, by age: Ages under 65 | | | 1963–75, by age: Ages 65 and over | | |
|---|---|---|---|---|---|---|---|---|---|
| *Mining towns* | Obs. | Exp. | SMR | Obs. | Exp. | SMR | Obs. | Exp. | SMR |
| Warsop | 24 | 20·08 | 120 | 4 | 4·13 | 97 | 9 | 10·14 | 89 |
| Mansfield Woodhouse | 32 | 34·66 | 92 | 8 | 7·03 | 114 | 16 | 17·78 | 90 |
| Kirkby in Ashfield | 38 | 42·39 | 90 | 6 | 7·52 | 80 | 29 | 22·40 | 129 |
| Sutton in Ashfield | 85 | 84·10 | 101 | 8 | 15·29 | 52 | 58 | 43·99 | 132* |
| Worksop | 81 | 65·70 | 123 | 12 | 11·73 | 102 | 45 | 34·59 | 130 |
| Mansfield | 116 | 115·36 | 101 | 26 | 20·04 | 130 | 54 | 60·93 | 89 |
| All mining towns | 376 | 362·29 | 104 | 64 | 65·74 | 97 | 211 | 189·83 | 111 |
| *Non-mining towns* | | | | | | | | | |
| Arnold | 38 | 53·20 | 71* | 5 | 9·32 | 54 | 25 | 28·81 | 87 |
| East Retford | 26 | 38·78 | 67* | 1 | 5·94 | 17* | 15 | 21·14 | 71 |
| Newark | 60 | 51·69 | 116 | 10 | 8·29 | 121 | 31 | 28·17 | 110 |
| West Bridgford | 53 | 62·19 | 85 | 5 | 8·55 | 58 | 34 | 35·00 | 97 |
| All non-mining towns | 177 | 205·86 | 86* | 21 | 32·10 | 65 | 105 | 113·12 | 93 |

* $P < 0{\cdot}05$.

described here in outline; a more detailed account is contained in an Appendix available on request.

RESULTS

Tables IV and V give the results for men and women, standardized for social class and mining. In the mining towns mortality is generally somewhat low among men; SMRs are relatively lower overall among men aged 65 and over than among younger men, but there is no consistent pattern in the 6 towns, and some SMRs based on small numbers are unstable. Mortality is slightly raised overall among women, especially among those aged 65 and over, but again there is no consistent pattern. For Worksop the results for men are unremarkable; mortality is raised among women, but not significantly so, and the SMR at ages 65 and over is virtually the same as in Kirkby and Sutton.

In the non-mining towns mortality is generally low (except in Newark) especially among women, and particularly in both sexes at ages under 65.

DISCUSSION

*Mortality in the 6 mining towns*

Although excess stomach cancer mortality among miners and their wives in England and Wales has been clearly documented (RG, 1958*a*, 1971; OPCS, 1978) the only published data on the overall mortality from this disease in mining towns relates to county boroughs (RG, 1958*b*, 1967; OPCS, 1979). However, the proportion of miners is rarely very high in such large towns, even if they are situated in the heart of mining districts; the proportion may be higher in smaller and more homogeneous towns, and when 27–47% of occupied men are miners, as in the 6 Nottinghamshire mining towns, one would expect stomach cancer mortality among men to show an overall rise. Without standardization for social class and mining, the overall SMR for men in the 6 towns during 1958–75 is 103 (based on O/E figures of 533/519·53), only slightly above the national SMR of 100, but 8% above the average regional SMR of 95. Standardization reduces the SMR to just below the regional average at 92, a result consistent with the hypothesis that stomach-cancer mortality is normal among male residents of these towns, except among miners and ex-miners.

For women the overall SMR in the mining towns is 114 ($P \sim 0{\cdot}01$ based on O/E figures of 376/328·50), significantly above national and regional SMRs. Standardization reduces the SMR to 104, and bearing in mind that all the results are approximate, this SMR should probably be taken to indicate that mortality is not generally raised among women in these towns. It should be remembered that the accuracy of the estimated SMR of 138 for Nottinghamshire miners' wives cannot be assessed, and that in any case it is not known why miners' wives have stomach-cancer SMRs as high as those of their husbands, nor how wide a range of women sharing mining households for part of their lives are affected by the factor(s) responsible.

Using the 5% level of significance, at least one SMR might differ "significantly" from 100 by chance among the 24 independent O/E comparisons by age for 1963–75 in the mining towns; in fact Tables IV and V show that by this criterion one SMR is "significantly high" and one is "significantly low". The raised SMR for women aged 65 and over in Sutton should be viewed alongside the low SMR at younger ages and the normal SMR over the whole period 1958–75, and could be due to chance; on the other hand the raised SMR is in line with those for Kirkby and Worksop. The low SMR for men at ages 65 and over in Warsop is perhaps of more interest, for among men (but not women) mortality is low in this town over the whole period; Warsop has the highest proportion of miners and the least-favourable class distribution, and is also by far the smallest of the mining towns.

*Mortality in the non-mining towns*

In these 4 towns the adjustment factors relate only to social class, and in Arnold, East Retford and Newark their effect is minimal. In West Bridgford, however, their effect is marked, because this town has an unusually favourable class distribution: the unadjusted SMRs are 71 ($P < 0{\cdot}01$) for men and 74 ($P < 0{\cdot}05$) for women, but after adjustment these rise to 82 and 85.

In the aggregate of non-mining towns the adjusted SMR of 91 for men of all ages during 1958–75 is very close to that for the aggregate of mining towns (92); West Bridgford has the lowest SMR (82), followed by East Retford (86). However, in contrast to the mining towns, mortality during 1963–75 is near-normal at ages 65 and over but significantly low at younger ages (SMR 74, $P < 0{\cdot}05$) and this trend is consistent in each town.

Among women, SMRs for 1958–75 are markedly low except in Newark, and at ages under 65 during 1963–75 are strikingly reduced, except again in Newark. It is interesting to compare the 1958–75 SMRs for East Retford and Newark: they are 67 (95% confidence limits 42–98) and 116 (89–149); superficially these two towns appear similar, but East Retford is smaller and less industrial. Mortality in the aggregate of non-mining towns is not greatly reduced at ages 65 and over (SMR 93) but is still much more favourable than equivalent mortality in the mining towns (SMR 111). It seems clear that the relatively favourable class structure of the non-mining towns cannot alone explain the lower stomach-cancer mortality among women of all ages and among men aged under 65.

The two age-groups analysed (under 65, 65 and over) have not been subdivided hitherto because of small numbers, but the results for ages under 45 are of particular interest. In the aggregate of mining towns the O/E figures are unremarkable at 8/10·05 for men and 6/5·91 for women. By contrast, in the non-mining towns the equivalent figures are 1/4·76 and 0/2·85, totalling 1/7·61. Thus the favourable stomach-cancer mortality in these 4 towns is most marked at ages under 45, and least so at ages 65 and over.

*Mortality in Worksop*

The final adjusted result for stomach-cancer mortality among men resident in Worksop during 1958–75 (SMR 97) clearly differs from Hill *et al.*'s result for 1963–71 (SMR 108). Table I shows how the SMR is reduced to 97, giving separate adjustments for social class and mining. The use of more accurate populations and the adjustment for social class make small differences, but the major contribution comes from the adjustment for mining. As shown in Estimate (6) the doubling of the length of the study period has little effect, and actually causes a slight rise in SMR.

For women the difference in the results is more striking: Hill *et al.*'s 1963–71 SMR was 160 ($P < 0{\cdot}01$) whereas our final adjusted SMR for 1958–75 is 123, no longer significantly raised. Table I shows that the major contributions to the reduction come from the use of more accurate populations and from the adjustment for mining, but that the doubling of the length of the study period also plays an important part. It happened that stomach cancer mortality among women resident in Worksop was higher during 1963–71, with an adjusted SMR of 131, than in either 1958–62 when the SMR was 124 (based on 24 deaths), or in 1972–75 when the SMR dropped to 104 (based on 14 deaths).

Hill *et al.* concluded that "compared with low nitrate control towns, Worksop has an increased death rate from gastric cancer". The results of the present study show that if allowance is made for social class structure and numbers of miners there is little indication that Worksop has a higher death rate than the other 5 nearest mining towns in Nottinghamshire. If the results shown in Tables IV and V were examined without any prior hypotheses concerning individual towns, it is doubtful whether Worksop would be singled out for attention. There does not appear to be any firm basis for suggesting that the high level of nitrate in the drinking water of Worksop has raised stomach-cancer death rates among its residents.

Miss J. Miller and Miss J. Adams assisted at various stages of this study. The Office of Population Censuses and Surveys made available unpublished data on numbers of deaths. The Institute of Cancer Research receives support from the Medical Research Council and the Cancer Research Campaign.

### REFERENCES

CASE, R. A. M. (1976) *Serial Mortality Tables: Neoplastic Diseases Volume I: England and Wales 1911–1970*. London: Institute of Cancer Research.

CHILVERS, C. & ADELSTEIN, A. (1978) Cancer mortality: The regional pattern. *Population Trends*, **12**, 4.

GENERAL REGISTER OFFICE (1954) *Census 1951: County report, Nottinghamshire*. London: H.M.S.O.

G.R.O. (1956*a*) *Census 1951: Occupation tables*. London: H.M.S.O.

G.R.O. (1956*b*) *Census 1951: Report on usual residence and workplace*. London: H.M.S.O.

G.R.O. (1964) *Census 1961: County report, Nottinghamshire*. London: H.M.S.O.

G.R.O. (1966*a*) *Census 1961: Occupation tables*. London: H.M.S.O.

G.R.O. (1966*b*) *Census 1961: Occupation, industry, socio-economic groups, Nottinghamshire*. London: H.M.S.O.

G.R.O. (1967) *Sample census 1966: County report, Nottinghamshire*. London: H.M.S.O.

G.R.O. (1968) *Sample census 1966: Economic activity county leaflets: general explanatory notes*. London: H.M.S.O.

HEWITT, D. (1956) Vagaries of local mortality rates under the 1953–54 rules for transfer of deaths. *Br. J. Prev. Soc. Med.*, **11**, 45.

HILL, M. J., HAWKSWORTH, G. & TATTERSALL, G. (1973) Bacteria, nitrosamines and cancer of the stomach. *Br. J. Cancer*, **28**, 562.

OFFICE OF POPULATION CENSUSES AND SURVEYS (1972) *Social survey division: A quality check on the 1966 ten per cent sample census of England and Wales*. London: H.M.S.O.

O.P.C.S. (1973) *Census 1971: County report, Nottinghamshire, Part I*. London: H.M.S.O.

O.P.C.S. (1975*a*) *The registrar general's revised estimates of the population of England and Wales 1961 to 1971*. London: H.M.S.O.

O.P.C.S. (1975*b*) *Census 1971: Economic activity county leaflet, Nottinghamshire*. London: H.M.S.O.

O.P.C.S. (1977) *Monitor Ref. PP1, 77/3*. London: H.M.S.O.

O.P.C.S. (1978) *Occupational mortality 1970–1972, England and Wales* (Chapter 8). London: H.M.S.O.

O.P.C.S. (1979) *Area mortality 1969–1973 tables. Series DS No.* 3 (microfiche) London: O.P.C.S.

REGISTRAR GENERAL (1958*a*) *Decennial supplement, England and Wales 1951: occupational mortality, Part II*. London: H.M.S.O.

R.G. (1958*b*) *Decennial supplement, England and Wales 1951: area mortality*. London: H.M.S.O.

R.G. (1960 *et seq.*) *Statistical review of England and Wales, 1958 et seq., Part III Commentary*. London: H.M.S.O.

R.G. (1967*a*) *Decennial supplement, England and Wales 1961: area mortality tables*. London: H.M.S.O.

R.G. (1967*b et seq.*) *Statistical review of England and Wales, 1965 et seq., Part I Tables Medical*. London: H.M.S.O.

R.G. (1971) *Decennial supplement, England and Wales 1961: occupational mortality tables*. London: H.M.S.O.

WORLD HEALTH ORGANISATION (1948) *Manual of the international statistical classification of diseases, injuries, and causes of death*, 6th Revision. Geneva: W.H.O.

W.H.O. (1957) *Manual of the international statistical classification of diseases, injuries, and causes of death*, 7th revision. Geneva: W.H.O.

W.H.O. (1967) *Manual of the international statistical classification of diseases, injuries, and causes of death*, 8th Revision. Geneva: W.H.O.